# Association of Tumor Necrosis Factor Receptor 1 Promoter Gene Polymorphisms (-580 A/G and -609 G/T) and TNFR1 Serum Levels with the Susceptibility to Gastric Precancerous Lesions and Gastric Cancer Related to *H. pylori* Infection in a Moroccan Population

**DOI:** 10.1155/2020/2451854

**Published:** 2020-09-24

**Authors:** Ghizlane Bounder, Mohamed R. Jouimyi, Hasna Boura, Hassan Jouhadi, Wafaa Badre, Hakima Benomar, Anass Kettani, Halima Lebrazi, Fatima Maachi

**Affiliations:** ^1^Helicobacter Pylori and Gastric Pathologies Laboratory, Institut Pasteur du Maroc, Casablanca 20360, Morocco; ^2^Biology and Health Laboratory, Faculty of Sciences Ben M'sik, University Hassan II, Casablanca 7955, Morocco; ^3^Department of Radiotherapy Oncology, Ibn Rochd University Hospital Center, Casablanca 20250, Morocco; ^4^Gastroenterology Department, Ibn Rochd University Hospital Center, Casablanca 20250, Morocco; ^5^Histo-Cytopathology Laboratory, Institut Pasteur du Maroc, Casablanca 20360, Morocco

## Abstract

Chronic inflammation due to *H. pylori* infection is the risk factor of gastric cancer (GC). Through its receptor (TNFR1), TNF-*α* plays a fundamental role in inflammatory, infectious, and tumor processes. Dysregulation of TNFR1 gene expression could impact many biological processes that can lead to cancer. This study is aimed at evaluating the association of TNFR1 promoter gene polymorphisms (-580 A/G and -609 G/T) and TNFR1 serum levels with GC and precancerous lesion susceptibility. Patients suffering from gastric lesions (65 chronic gastritis, 50 precancerous lesions, and 40 GC) related to *H. pylori* infection and 63 healthy controls (HC) were involved in this study. Individuals are genotyped by TNFR1 gene promoter sequencing, and TNFR1 serum levels were measured by the ELISA quantitative method. Concerning TNFR1 -609 G/T locus, we noticed that the T allele was associated with an attenuated susceptibility to GC (OR = 0.4; *p* value = 0.02). At the genotypic level and under the recessive model, the TNFR1 -609 TT genotype showed a decreased risk of GC (OR = 0.3, *p* value = 0.03) compared to the combined (GG/GT) genotypes. TNFR1 serum levels have been increased together with gastric lesion severity (*p* value < 0.05). The TNFR1 -609 TT genotype seemed linked to a low level of sTNFR1 compared to GT and GG genotypes (*p* value = 0.07). Concerning TNFR1 -580 A/G locus, no significant relation was noticed between this polymorphism and GC susceptibility, as well as with the TNFR1 serum level. Our results suggest that the TNFR1 -609 T allele appears to have a protective effect against GC. High levels of TNFR1 serum levels seemed to be associated with the aggressiveness of gastric lesions. Therefore, our results suggest that TNFR1 -609 T/G polymorphism and the TNFR1 serum levels may be related to GC susceptibility.

## 1. Introduction

Gastric cancer is one of the most aggressive neoplasms. It is ranked among the fourth most diagnosed cancers and the second leading cause of death worldwide [1]. Gastric cancer is a multistep process, starting by *Helicobacter pylori-* (*H. pylori-*) driven inflammation, which leads to chronic gastritis, atrophic gastritis, intestinal metaplasia, and dysplasia which can then progress to an adenocarcinoma [2].

Chronic inflammation related to *H. pylori* infection is believed to be a critical step in gastric carcinogenesis [3]. Tumor Necrosis Factor-alpha (TNF-*α*) is known as a principal mediator of inflammation and host response to *H. pylori* infection, and it is closely related to gastric epithelial injury [4, 5]. Controversial functions have been attributed to this cytokine in cancer physiopathology. TNF-*α* can act as a tumor suppressor through vascular destruction, tumor necrosis, and immunostimulation. Or act as a tumor promoter by inducing cellular transformation, survival, proliferation, invasion, angiogenesis, and metastasis [6, 7]. TNF-*α* elicits its pleiotropic cellular effects through interaction with its two cell surface receptors TNFR1 and TNFR2. TNFR1 represents the primary receptor for TNF-*α* due to its ubiquitous expression and its enrolling in several biological effects [8].

The TNFR1 gene is located on chromosome 12p13 consisting of 10 exons, 9 introns, and a 3′ untranslated region. The TNFR1 gene contains a housekeeping promoter with multiple transcription start sites and CAAT box motifs and missing TATA box motifs [9]. The expression of TNFR1 is tightly regulated, in part, at the transcriptional level. Polymorphisms occurring in promoter regions of the TNFR1 gene may potentially affect the expression of TNFR1 [9]. Several studies have described some TNFR1 gene polymorphisms as sensitivity markers of inflammatory disorders and cancers [10–14].

TNFR1 is primarily produced as a transmembrane protein (tmTNFR1) consisting of 455 amino acids (55 kDa) that gets released from the membrane by proteolytic cleavage, to generate a soluble form (sTNFR1). sTNFR1 plays an important role in the regulation of TNF-*α* activity. It can act as a TNF-*α* antagonist by competing for the interaction of TNF-*α* with tmTNFR1, or as an activator through stabilizing the trimer structure of TNF-*α* by preventing its dissociation into inactive monomers, and serving as a slow-release reservoir for TNF-*α* [15]. sTNFR1 can also function as a ligand for tmTNF-*α* and induce intracellular messages via reverse signaling [16]. The serum levels of sTNFR1 and its ligand TNF-*α* are highly regulated in diverse human cancers and are suggested to be an important determinant of growth and cancer progression [7, 15, 17–19].

The identification of predictive markers for the identification and follow-up of patients that present a high risk to develop gastric cancer is the main objective of several studies. The present study is aimed at evaluating the association of TNFR1 -580 A/G and TNFR1 -609 G/T polymorphisms and sTNFR1 serum levels with gastric cancer susceptibility.

## 2. Materials and Methods

### 2.1. Clinical and Pathological Characteristics of the Study Population

This retrospective study focused on patients suffering from gastric disorders associated with *H. pylori* infection, collected from the Gastroenterology and Oncology Departments at the IBN ROCHD University Hospital Center from 2016 to 2018. Patients having received previous treatment for *H. pylori* eradication, proton pump inhibitors, anti-inflammatory medicines, chemotherapy or radiotherapy treatment, or suffering from any other infections, inflammatory disorders, and cancers other than distal gastric adenocarcinoma were excluded from the study. Healthy asymptomatic subjects, with no history of gastrointestinal illnesses or regular use of any gastrointestinal and anti-inflammatory medicines, were recruited from the Regional Transfusion Center of Casablanca.

All participants were informed about their inclusion in the study and agreed to it in writing form. The study protocol has been performed in accordance with the ethical standards of Helsinki and was approved by the committee of Pasteur Institute of Morocco.

Three biopsies (1 antrum, 1 fundus, and 1 lesser curvature) were sampled from patients admitted for endoscopy. Blood samples have been sampled from all healthy controls and patients before anesthesia and gastroscopy examination and conserved in an icebox during transport.

The biopsies were fixed in 10% formalin and taken to the Histo-Cytopathology Laboratory in Pasteur Institute of Morocco, where they were embedded in paraffin blocks, sectioned and stained in hematoxylin and eosin for conventional histopathology examination of gastric mucosal lesions and *H. pylori* infection. Classification of gastric lesions was done according to the Sydney system.

The diagnosis of *H. pylori* infection in the healthy control subjects was performed by the quantitative anti-*H. pylori* IgG ELISA method, using a commercially available kit (Euroimmun, D-23560 Lubeck (Deutschland)). A cut-off value of antibody concentration ≥ 20 relative units (RU)/mL was considered as positive.

### 2.2. Genotyping of TNFR1 -609/-580 Polymorphisms

Genomic DNA was isolated from blood taken on the EDTA tube using the commercially available kit (PureLink™ Genomic DNA Mini Kit) and stored at −20°C until use.

Individuals were genotyped for TNFR1 gene promoter at position −609 G/T and −580 A/G by sequencing. Firstly, the TNFR1 gene (360 bp) was amplified from genomic DNA by PCR. The PCR was carried out in a total reaction volume of 20 *μ*L, consisting of 400 ng genomic DNA, 1 mM of dNTPs, 3 mM of MgCl2, 0.5 *μ*M of primers as described by Gupta et al. [11], and 1 unit of Taq polymerase (MyTaq^TM^ Polymerase, Bioline). Thermocycling conditions of PCR were as follows: initial denaturation at 95°C for 1 minute, followed by 30 cycles of 95°C for 15 seconds, 57°C for 15 seconds, 72°C for 30 seconds, and a final extension at 72°C for 7 minutes. The PCR products were checked out in 1.5% agarose gel electrophoresis and purified using 1 *μ*L of exonuclease (Thermo Scientific), 1 *μ*L of alkaline phosphatase (Promega), and 5 *μ*L of PCR product and incubated at 37°C for 15 minutes and 80°C for 15 minutes. The DNA sequencing conditions were carried out based on the method as previously described [19]. The DNA sequences were read by an Applied Biosystems 377 DNA sequencer and analyzed using the BIOEDIT software package.

### 2.3. Measurement of sTNFR1 Serum Levels

The blood samples were taken on the serum tube, and the serum was obtained by centrifugation at 1500 g for 15 min within 1 h of blood collection. The serum was stored at -80°C in aliquots of 500 *μ*L and melted just before testing. The measurement of sTNFR1 serum levels was performed in strict accordance with the kit instructions: the Human TNFR1 ELISA kit from R&D Systems (DuoSet ELISA, USA). Total sTNFR1 concentrations in samples were expressed as pg/mL.

### 2.4. Statistical Analysis

The statistical program R software version 3.5.0 was used to carry out the statistical analysis. The descriptive data were presented in terms of frequencies. A chi-square test was used for the comparisons of genotypes and allele frequencies between cases and controls. When the number of subjects in the subgroup was lower than 10, the Fisher's correction was used. Odds Ratios (OR), as well as their 95% confidence intervals (CI), were computed to estimate the risk of gastric lesions with TNFR1 variants. The normality of distribution was assessed by the Shapiro-Wilk test, and then, a comparison of quantitative data between two groups was performed by the Wilcoxon test. The comparison of quantitative data between more than two groups was performed by the Kruskal-Wallis test. The differences were regarded as significant at *p* value < 0.05.

## 3. Results

### 3.1. Clinical and Pathological Characteristics of the Study Population

A total of 218 participants with *H. pylori*-positive status, composed of 63 asymptomatic healthy control subjects (HC) and 155 patients with various gastric lesions, 65 with chronic gastritis, 50 with precancerous lesions (30 with atrophic gastritis, 20 with intestinal metaplasia), and 40 with gastric cancer, were enrolled in the present study. Twenty-four patients and 4 HC were excluded.

The male/female ratio in the case group and the control group was, respectively, 73/82 and 34/29. The mean age of HC was 43 ± 8, chronic gastritis was 45 ± 16, precancerous lesions was 53 ± 14, and gastric cancer was 56 ± 14 years.

### 3.2. Genotyping of TNFR1 -609/-580 Polymorphisms

Regarding TNFR1 −609 G/T locus, the frequency of T allele is 37.3% in HC and has shown a decrease with the severity of gastric lesions, with the following order: 36.2% in chronic gastritis, 31% in precancerous lesions, and 21.3% in gastric cancer. The T allele was observed to be associated with an attenuated risk of gastric cancer with an OR = 0.4 (*p* value = 0.02) compared to the G allele (Table 1). Genotypically, we noticed that the TT genotype was lower in patients with gastric cancer (10%) and precancerous lesions (16%) compared to patients suffering from chronic gastritis (23.1%) and HC (28.6%). But statistically, the alteration remains not significant (*p* value = 0.07). According to the different dominance models of the TNFR1 -609 T allele, we noticed that in cases of dominance and codominance, the combined genotypes (TT+GT) reduced the risk of gastric cancer, but statistically, the results were not significant (OR = 0.5, 95% CI 0.2-1.2, *p* value = 0.2). While under the recessive model, we noticed a significantly reduced risk for gastric cancer (OR = 0.3, 95% CI 0.09-0.9, *p* value = 0.03) in subjects with the TNFR1 -609 TT genotype compared to the combined genotypes (GG+GT). The distribution of alleles and genotypes for the TNFR1 −609 G/T locus in the patient groups and controls is shown in Table 1.

Regarding the TNFR1 −580 A/G locus, there were no significant differences in allele and genotype distributions between patients and HC. The distribution of alleles and genotypes for the TNFR1 −580 locus in the patient groups and controls is shown in Table 1.

### 3.3. Measurement of sTNFR1 Serum Levels

The serum levels of sTNFR1 were observed to be increased together with the severity of gastric lesions. The lowest levels were recorded in controls with an average value of 779.8 pg/mL and have shown an increase with the severity of gastric lesions, with the following order: 881.3 pg/mL in patients with chronic gastritis, 892.6 pg/mL in those with precancerous lesions, and 929.9 pg/mL recorded among patients suffering from gastric cancer (Table 2).

A disparity in the sTNFR1 serum levels between all the groups of patients and HC was noted and was statistically significant (*p* value < 0.05). However, no significant difference was observed between chronic gastritis, PL, and gastric cancer (Table 2).

### 3.4. Association between TNFR1 -609/-580 Polymorphisms and sTNFR1 Serum Levels

Regarding the TNFR1 -609 G/T locus, the mean level of sTNFR1 in GG (872.1 pg/mL) and GT (875.6 pg/mL) genotypes was approximately the same, while a relatively low value of sTNFR1 was observed in the TT genotype (820.4 pg/mL) (Table 3). It appears that individuals with the TT genotype tend to express low sTNFR1 levels compared to those with GT and GG genotypes, but the difference remains statistically not significant (*p* value = 0.07).

Regarding the TNFR1 -580 A/G locus, no difference in the distribution of sTNFR1 serum levels according to the genotypes was revealed (Table 3). The mean level of sTNFR1 was, respectively, 866.2, 852.5, and 826.71 pg/mL in individuals with AA, GA, and GG genotypes.

## 4. Discussion

The genetic predisposition to cancers has been demonstrated through several studies and is still a subject of interest and scientific research [11, 13, 19]. Serum levels of sTNFR1 are used as a predictive marker of multifactorial inflammatory disorders and cancers [12, 17, 20, 21]. The present study was designed to evaluate the potential use of TNFR1 genotyping (TNFR1 -580 A/G and TNFR1 -609 G/T) and TNFR1 serum levels as predictive markers in the identification and follow-up of patients that present a high risk to develop gastric cancer.

The association of TNFR1 -609 G>T and TNFR1 -580 A>G polymorphisms with the susceptibility to gastric cancer and precancerous lesions in our population was evaluated.

Concerning the TNFR1 -609 G/T locus, it seemed that the frequency of the T allele and TT genotype decreased with the severity of the gastric lesions. The T allele was observed to be associated with an attenuated risk of gastric cancer with an OR = 0.4 (*p* value = 0.02) compared to the G allele. The TNFR1 -609 TT genotype was associated with a reduced risk of gastric cancer (OR = 0.3, 95% CI 0.09-0.9, *p* value = 0.03) compared to the combined genotypes (GG+GT), under the recessive model.

Our finding is consolidated with several studies that reported the association of this polymorphism with a reduced risk of oral, colon, and gastric cancer and other inflammatory pathologies such as invasive pulmonary aspergillosis [10–13]. However, other reports have shown the association of TNFR -609 T allele with a high risk of hepatic cancer, Crohn's, and ankylosing spondylitis diseases [22–24]. The effect of this polymorphism on cancer pathologies is still unknown, so further studies are needed to clarify this association.

Concerning the TNFR1 -580 A/G locus, statistically, no alterations in the genotypic and allelic distributions between groups were noted (*p* value > 0.05). This result is consolidated by other studies reporting the absence of the association between TNFR1 -580 A/G polymorphism and gastric pathologies, chronic pancreatitis, rheumatoid arthritis, and Kawasaki disease [10, 25, 26].

Determination of sTNFR1 levels in several body fluids, including serum, provides valuable information on a variety of pathological conditions [27]. In this study, a significant disparity of sTNFR1 serum levels between cases and controls was noticed. Compared to HC, we remarked that sTNFR1 serum levels increase among patients with chronic gastritis, precancerous lesions, and mainly among those suffering from gastric cancer. Our results suggest a positive association between the increase of sTNFR1 serum levels and gastric carcinogenesis.

*H. pylori* infection provokes the production of inflammatory mediators, including TNF-*α*, IL8, IL1, MMPs, and growth factors [28, 29]. These mediators can activate the expression of the TNFR1 gene and its release [16, 21]. Shibata et al. showed an elevation of sTNF-*α* and sTNFR1 in *H. pylori*-infected patients compared to uninfected patients [30]. Regarding cancer diseases, increased levels of circulating sTNFR1 have been reported in patients suffering from several cancers [7, 15, 27]. As regards gastric cancer, Shibata et al. revealed that high serum levels of sTNFR1 were associated with the severity of gastric cancer (studied population: 42 gastric cancer patients compared to 15 HC) [31]. Aderka et al.'s study showed that TNFR1 serum levels were higher in patients with gastric cancer than those in controls (studied population: 3 patients compared to 42 HC) [32]. Several studies suggest that high levels of sTNFR1 in patients are associated with poor prognosis, more aggressive immunological response, high severity of cancer, advanced tumor stages, a reduced response to treatment, high mortality, and short survival [7, 17, 20, 21, 33, 34], while other studies reported that a high level of sTNFR1 reflects the antitumor defense and could be used to estimate the immune potential of cancer patients [35, 36].

The polymorphisms located in the promoter region of the TNFR1 gene could have a significant impact on transcriptional regulation. Our findings revealed that individuals carrying TNFR1 -609 TT genotype tend to express low sTNFR1 levels compared to those carrying TNFR1 -609 GT and GG genotypes, but these observations remain statistically insignificant (*p* value = 0.07). Previous studies have reported the repression impact of this polymorphism on TNFR1 expression [9, 23]. TNFR1 -609 T allele was associated with an attenuated risk of the gastric cancer group and was linked to low TNFR1 levels. We can suggest that the elevation of circulating sTNFR1 is implicated in the promotion of gastric cancer and not in its regression. Regarding the TNFR1 -580 A/G locus, no alteration in sTNFR1 serum levels according to the genotypes of this locus was recorded. This finding is supported by Wang et al. who demonstrated the absence of the impact of TNFR1 -580 A/G polymorphism on TNFR1 production [37]. Further research on a larger population is needed to clarify the effect of these polymorphisms.

## 5. Conclusions

In summary, the TNFR1 -609 T allele seems to be linked to an attenuated susceptibility to gastric cancer. A gradual increase in serum levels of sTNFR1 with the severity of gastric carcinogenesis lesions was demonstrated. TNFR1 -609 TT genotype appears to be associated with reduced sTNFR1 serum levels. Regarding the TNFR1-580 A/G locus, no significant association with a gastric cancer risk or its impact on sTNFR1 serum levels was recorded. Further studies including a large population and diverse ethnicities are needed to illumine the association of TNFR1 -609 G/T and -580 A/G polymorphisms and sTNFR1 serum levels with gastric cancer susceptibility, to be used as potential markers for the identification and follow-up of patients at a high risk of developing gastric cancer.

## Figures and Tables

**Table 1:** TNFR1 promoter polymorphisms at positions -609 and -580 in patients with gastric lesions and healthy controls.

	HC	CG	PL	GC
	*N* (%)	*N* (%)	*N* (%)	*N* (%)
TNFR1 -609 G/T genotypes				
GG	34 (54.0)	33 (50.8)	27 (54)	27 (67.5)
GT	11 (17.4)	17 (26.1)	15 (30)	9 (22.5)
TT	18 (28.6)	15 (23.1)	8 (16)	4 (10.0)
*p* value^∗^		0.47	0.15	0.07
OR, [95% CI], *p* value (TT+GT vs. GG)		1, [0.5-2.1], 0.86	0.9, [0.42-1.9], 0.8	0.5, [0.2-1.3], 0.2
OR, [95% CI], *p* value (GG+GT vs. TT+GT)		0.9, [0.5-1.9], 1	0.8, [0.4-1.7], 0.7	0.5, [0.2-1.2], 0.2
OR, [95% CI], *p* value (GG+GT vs. TT)		0.7, [0.3-1.6], 0.5	0.4, [0.1-1.2], 0.2	0.3, [0.09-0.9], 0.03
TNFR1 -609 G/T alleles				
G	79 (62.7)	83 (63.8)	69 (69)	63 (78.7)
T	47 (37.3)	47 (36.2)	31 (31)	17 (21.3)
*p* value^∗^		0.85	0.32	0.01
OR, [95% CI], *p* value (T vs. G)		0.9, [0.6-1.6], 0.89	0.7, [0.4-1.3], 0.33	0.4, [0.2-0.8], 0.02
TNFR1 -580 A/G genotypes				
AA	45 (71.4)	55 (84.6)	33 (66)	33 (82.5)
AG	16 (25.4)	9 (13.8)	17 (34)	6 (15)
GG	2 (3.2)	1 (1.6)	0 (0)	1 (2.5)
*p* value^∗^		0.17	0.42	0.42
OR, [95% CI], *p* value (GA+GG vs. AA)		0.4, [0.2-1.1], 0.08	1.3, [0.6-2.9], 0.54	0.5, [0.2-1.4], 0.24
OR, [95% CI], *p* value (AA+GA vs. GG+GA)		0.5, [0.2-1.2], 0.1	1.1, [0.5-2.5], 0.8	0.6, [0.2-1.6], 0.36
OR, [95% CI], *p* value (AA+GA vs. GG)		0.5, [0.04-5.4], 0.6	0, [0-0], 0.5	0.6 [0.07-8.9], 1
TNFR1 -580 A/G alleles				
A	106 (84.1)	119 (93)	83 (83)	72 (90)
G	20 (15.9)	11 (7)	17 (17)	8 (10)
*p* value^∗^		0.08	0.82	0.23
OR, [95% CI]; *p* value (G vs. A)		0.5, [0.2-1], 0.08	1.1, [0.5-2.2], 0.85	0.6, [0.2-1.4], 0.29

HC: healthy controls; CG: chronic gastritis; PL: precancerous lesions; GC: gastric cancer. ^∗^*p* values were calculated using the chi-squared test or Fisher test.

**Table 2:** Association between sTNFR1 serum levels and gastric lesion severity.

sTNFR1 (pg/mL)	HC	CG	PL	GC
Mean	779.8	881.3	892.6	929.9
Median	780	881	921	881
SD	105.1	158	154.6	196.7
Range	540-958	622-1257	520-1132	633-1439
*p* value significance intragroups^∗^	0.000018			
*p* value significance intergroups^∗∗^				
Case vs. controls		0.0003	0.00003	0.0002
Case vs. chronic gastritis		—	0.36	0.28
Case vs. precancerous lesions		—	—	0.61

HC: healthy controls; CG: chronic gastritis; PL: precancerous lesions; GC: gastric cancer; ∗: Kruskal-Wallis test; ∗∗: Wilcoxon test.

**Table 3:** Association between TNFR1 -609 G/T and -580 A/G locus genotypes and sTNFR1 serum levels.

TNFR1 (pg/mL)	TNFR1 -609 G/T genotypes			TNFR1 -580 A/G genotypes		
	GG	GT	TT	AA	AG	GG
Range	520-1272	630-1251	540-1129	520-1272	680-1237	540-1112
Mean	872.1	875.6	820.4	866.2	852.5	826.7
SD	163.9	153.1	141.7	162.1	138.3	249.2
*p* value significance intragroups^∗^	0.15			0.85		
*p* value significance intergroups^∗∗^						
Case vs. TNFR1 -609 GG		0.97	0.07			
Case vs. TNFR1 -609 GT			0.07			
Case vs. TNFR1 -580 AA					0.65	0.83
Case vs. TNFR1 -580 AG						0.7

∗: Kruskal-Wallis test; ∗∗: Wilcoxon test.

## Data Availability

The data used to support the findings of this study are included within the article.
